# Effectiveness of simulation-based interprofessional education on teamwork and communication skills in neonatal resuscitation

**DOI:** 10.1186/s12909-024-05581-1

**Published:** 2024-05-31

**Authors:** Shinhye Chae, Soonyoung Shon

**Affiliations:** 1 College of Nursing, Keimyung University, 1095 Dalgubeol-daero, Dalseo-gu, Daegu, 42601 Republic of Korea; 2 Nursing department, Keimyung University Dongsan Hospital, Daegu, Republic of Korea

**Keywords:** Interprofessional education, Simulation, Neonate resuscitation, Nursing education

## Abstract

**Background:**

The role of effective interprofessional teamwork is especially vital in the Neonatal Intensive Care Unit (NICU) where infants facing emergency situations are admitted. Proper neonatal resuscitation, facilitated by comprehensive resuscitation training, can significantly decrease the mortality rates associated with neonatal asphyxia and respiratory failure. This study aimed to develop a simulation-based interprofessional education (IPE) programme for medical staff working in a nursery and NICU and to assess its effectiveness on teamwork, communication skills, clinical performance, clinical judgement, interprofessional attitudes, and education satisfaction.

**Methods:**

Through a demand survey, neonatal resuscitation was selected as the theme, and an IPE team comprised of one doctor and two nurses was formed. The education programme consisted of three sessions lasting a total of 140 min: two simulation exercises and one theoretical education session. Data were collected from 18 nurses working in the nursery and NICU and 9 doctors working in the paediatrics department.

**Results:**

A comparison of the metrics before and after applying simulation-based IPE programmes revealed teamwork (*Z*=-2.67, *p* = .008), communication skills (*Z*=-2.68, *p* = .007), clinical performance (*Z*=-2.52, *p* = .012), clinical judgement (*Z*=-4.52, *p* < .001), and interprofessional attitude (*Z*=-3.64, *p* < .001) to have significantly improved. Education satisfaction scores were 4.73 points on average out of a maximum of 5. The simulation-based IPE programme was effective in improving the teamwork, communication, and clinical performance of resuscitation teams, individual clinical judgement, and interprofessional attitude.

**Conclusions:**

Simulation-based IPE is effective for enhancing teamwork, team communication, clinical judgement skills, and clinical performance in neonatal resuscitation. This programme has the potential to contribute to the improvement of patient safety and the quality of neonatal care. Additional studies are needed to longitudinally examine the effects of the programme on patient safety and quality of neonatal care.

## Background

Healthcare quality depends on interprofessional cooperation and teamwork, which are subsequently dependent upon trust between professionals. Ineffective teamwork can have a negative impact on patient safety and treatment [1], while better collaboration between healthcare professionals reduces the occurrence of medical errors [2]. Regarding patient outcomes, information about the treatment plan and progress should be promptly communicated between the medical staff who handle newborns; hence, communication between the medical staff is an important factor that contributes to the efficiency and safety of work procedures [3]. Therefore, all healthcare professionals working on the front line should strive to facilitate communication and improve teamwork.

The role of effective interprofessional teamwork is especially vital in the Neonatal Intensive Care Unit (NICU) where infants facing emergency situations are admitted [4, 5]. Notably, 30% of error reports within the NICU are linked to issues related to communication and teamwork [6]. Emergency situations in children, compared with those in adults, are difficult to predict and cause rapid changes in clinical status [5]. Neonatal resuscitation requires effective teamwork and competent medical skills, and are the most important factors in the first chain of neonatal survival [7]. However, due to ineffective teamwork by resuscitation teams, 30% of resuscitation procedures are incorrectly performed or not performed at all [8]; the lack of communication during neonatal resuscitation negatively impacts neonatal survival by interrupting rapid decisions [9].

Although the number of neonatal deaths worldwide has decreased from 5 million in 1990 to 2.4 million in 2019, 47% of under-five deaths occurred in the neonatal period, with approximately one-third of those deaths occurring on the day of birth [10]. Neonatal asphyxia and respiratory failure are the leading causes of neonatal mortality. Providing correct neonatal resuscitation through resuscitation training can reduce the neonatal mortality caused by neonatal asphyxia and respiratory failure [11]. To enhance the survival rates of newborns, it is imperative to have both skilled medical professionals and access to high-quality treatment [10]. Effective neonatal resuscitation requires the collaborative training of healthcare workers from diverse professional backgrounds [12]. In 2010, the World Health Organization (WHO) defined interprofessional education (IPE) as “supporting effective collaboration between two or more professional groups by learning with, learning from, and learning about each other, and it was made to improve the health outcomes” [13]. Interprofessional collaboration can prevent cross-professional conflict, improve leadership, and ultimately improve patient safety [14].

Therefore, this study aimed to develop and apply a simulation-based training programme on neonatal resuscitation for doctors and nurses and to examine their interprofessional teamwork experience, communication skills, clinical performance, clinical judgement, changes in attitude, and educational satisfaction.

## Methods

### Purpose and design

This study developed a simulation-based IPE programme for doctors and nurses working in the nursery of NICU, and evaluated the programme’s effect on teamwork, communication skills, clinical performance, clinical judgement, interprofessional attitudes, and education satisfaction. A pre-experimental single-group pretest-posttest design was conducted to evaluate its effectiveness.

### Sample and setting

The number of participants was calculated using G*power version 3.1.9.7; when a significance level of 0.05, an effect size of 0.50, and a power of 0.80 were set to compare the effect difference of a single group, the minimum number of samples required was 27. The inclusion criteria for participants were paediatricians and nurses specialising in neonatal care, specifically those employed in neonatal wards and neonatal intensive care units. Participation was voluntary and contingent upon a thorough comprehension of the research objectives. Nurses who were not directly engaged in patient care were excluded. The study included paediatricians and neonatal unit or NICU nurses working in K University Hospital in D Metropolitan City, South Korea. Data collection was conducted from December 1, 2021, to March 10, 2022, with recruitment and research conducted concurrently to accommodate participants’ schedules. As 27 participants were recruited without any dropouts during this process, further recruitment was halted, establishing a final research participant count of 27.

### Intervention

#### Preparation

The subject of the simulation-based IPE programme (SimIPE) was ‘neonatal resuscitation’, and this subject was chosen through a demand survey. The educational goals were set according to Bloom’s taxonomy [15]. The cognitive domain was aimed at explaining the necessity and importance of IPE, postnatal treatment and care, and neonatal resuscitation, while the affective domain was aimed at providing information related to interprofessional cooperative practice. Meanwhile, the psychomotor domain involved the performance of appropriate practice according to the sequence and situation immediately after birth, the application of respiratory support interventions, and the use of effective teamwork and communication skills.

#### Implementation

**Fig. 1:**
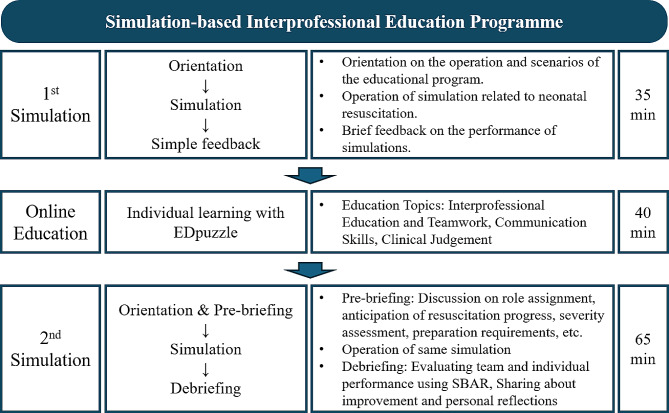
The programme content for simulation based interprofessional education

The programme comprised simulation practice and theory learning. The main content aimed at improving teamwork and communication skills as well as enhancing clinical judgement related to the appropriate therapeutic actions for neonatal resuscitation. The SimIPE was conducted in three sessions (Fig. 1). The first session took 35 min and included orientation to the components of the simulation environment and the first activity in the simulation, a preliminary survey to evaluate the level of clinical judgement and interprofessional attitude, and provision of simple simulation performance feedback. The second session consisted of three rounds of online education on how to achieve good interprofessional teamwork, interprofessional communication skills, and clinical judgement during neonatal resuscitation. Each round of online education took 40 min, and the entire second session was conducted for 2 weeks before the second simulation. Moreover, the participation rate was determined by asking the participants to answer the EDpuzzle quiz; all 27 participants completed the course. The quiz comprised nine questions related to the neonatal resuscitation process, and the overall response rate was 93%. The third session was conducted after more than 2 weeks from the first round of training to prevent the test effect from affecting the results of the third session. The third session consisted of orientation and prebriefing, the second simulation and debriefing (which took 65 min), and a posttest to evaluate the participants’ clinical judgement skills and interprofessional attitudes. The overall duration of the first three sessions was 140 min.

**Fig. 2:**
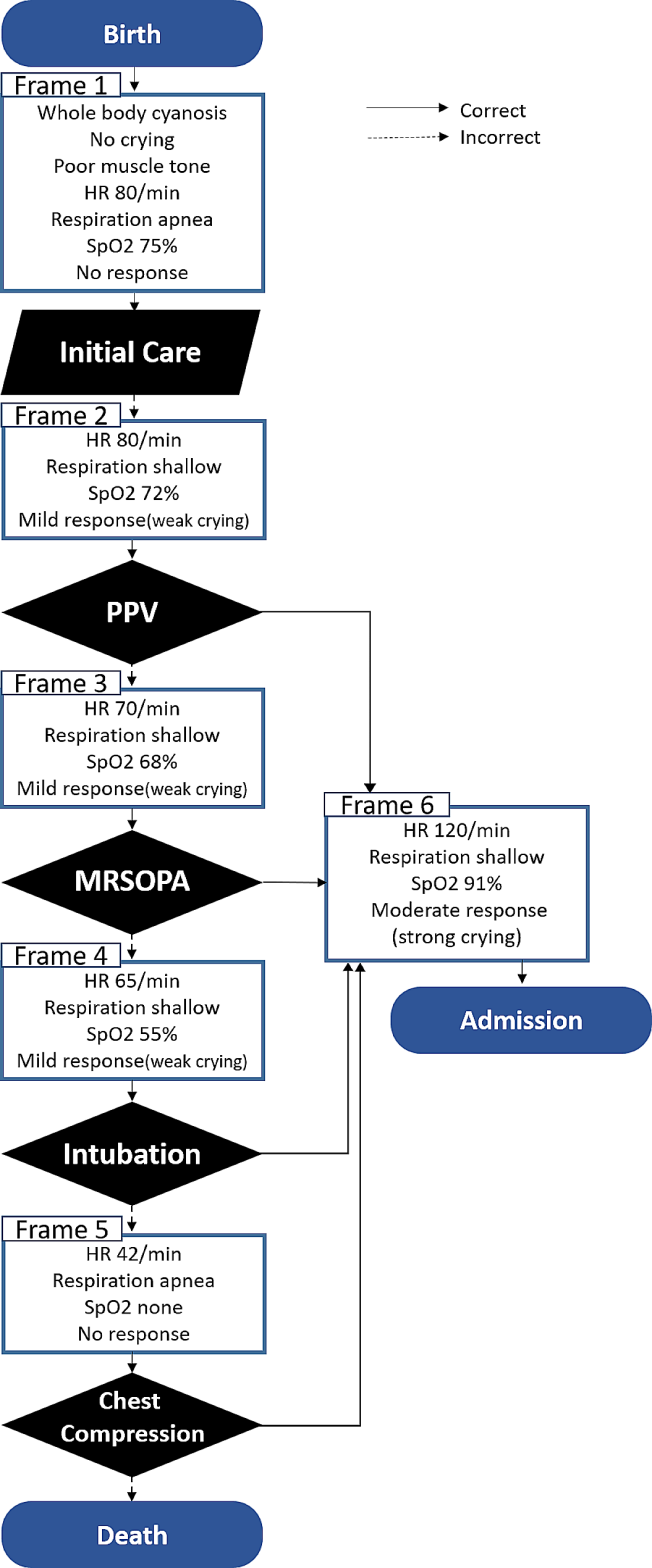
The algorithm of neonatal resuscitation for simulation based interprofessional education

The algorithm presented a series of procedures that the healthcare professionals were required to perform according to the existing neonatal resuscitation guidelines. The simulation scenario was structured to provide neonatal resuscitation for an extremely preterm, 25-week-old, very low birth weight infant, and involved a team consisting of one physician and two nurses. The doctor took on the role of the team leader and assigned specific roles to the nurses. One nurse was assigned tasks such as attaching monitors and suctioning, while another nurse was given responsibilities such as timing and monitoring checks. In the algorithm, the expected changes in the simulator’s response, heart rate, and oxygen saturation and the procedures that the learners were required to perform were presented according to the predetermined sequence (Fig. 2). In the initial care phase, the medical team performs activities such as warming the infant, attaching electrocardiogram electrodes and oxygen saturation sensors, measuring time, and providing stimulating cycles. Subsequently, positive pressure ventilation (PPV) is administered. If PPV proves ineffective, the medical staff proceeds with MRSOPA (mask adjustment, repositioning of the airway, suctioning of the mouth and nose, opening of the mouth, pressure increase, and airway alternative). If there is no response to MRSOPA, chest compressions are initiated following intubation. Once urgent intervention is completed, the infant receives finish care such as hospitalisation.

The questions used for debriefing were formulated following the Gather-Analysis-Summarise (GAS) model developed by the WISER simulation centre of the University of Pittsburgh Medical Center. The debriefing session lasted for 30 min; a situation-background-assessment-recommendation (SBAR) report was prepared, and the content was compared with the results of other participants. The SBAR technique is one of the standardised communication methods that are easy to perform and enables the collection of concise information; it also allows sufficient sharing of the collected information with other people involved. After SBAR reporting, the team watched the simulation they conducted on video and individually noted areas for discussion. Detailed feedback regarding the learners’ simulation performance was provided, and discussions were facilitated using questions formulated based on the GAS model. For example, discussions were held regarding how each participant performed their roles, what went well, areas for improvement, and how they felt about their participation.

#### Evaluation of outcomes

After implementing the SimIPE, the educational outcomes were analysed. Simulation performance, teamwork, and communication skills were evaluated by two instructors who reviewed recorded videos. Clinical judgement and interprofessional attitudes were assessed using self-reported surveys completed by the participants. For accurate effectiveness evaluation and to provide feedback during debriefing sessions, two instructors observed the simulation process to assess clinical judgement. Finally, satisfaction with the educational programme was surveyed.

### Instruments

#### Teamwork

To assess the level of teamwork, the Simulation Team Assessment Tool [STAT] developed by Reid et al. [16] was initially translated, then back-translated, and subsequently evaluated for expert validity. The tool was translated in accordance with the WHO guidelines on Translation and Interpretation of Instruments [17]; the translation was reviewed for semantic equivalence, consistency, accuracy, and context by one professor of paediatric nursing and two nurses with a master’s degree or higher and at least 5 years of experience working in a NICU.

The STAT was developed to assess the level of teamwork and performance during paediatric resuscitation; this instrument comprises 94 items divided into the following four domains: basic assessment skills, airway/breathing, circulation, and human factors. Each item is rated on a 3-point scale: 1 as ‘complete and timely’, 2 as ‘incomplete or untimely’, and 0 as ‘needed and not done’. The total score ranged from 0 to 188, and a higher score indicated greater teamwork and performance. As the techniques used in performing paediatric and neonatal resuscitations differ, only the human factor domain was employed to measure teamwork in this study. This domain consists of 26 items that are used to evaluate the whole team, leader, and team members, and the total score ranges from 0 to 52. The intraclass correlation coefficient (ICC) values were 0.81 at the time of development and 0.80 in this study.

#### Communication skills

Communication skills were assessed using the Indiana University Simulation Integration Rubric (IUSIR) tool developed by Reising et al. [18] after translating, back-translating, and establishing the expert validity of the tool. The IUSIR was also translated in accordance with the WHO guidelines on Translation and Interpretation of Instruments [17].

The IUSIR tool was developed to evaluate the communication skills of healthcare professionals; it comprises 12 items that are divided into two domains: individual and team. Each item is rated on a three-point scale: 1 as ‘below average’, 3 as ‘average’, and 5 as ‘above average’. Scores between 2 and 4 were assigned as necessary. The scores for the individual and team domains each ranged from 6 to 36, and a higher score indicated better communication skills. As neonatal resuscitations were the focus of this study, we excluded items that assessed the healthcare professional-patient interactions; hence, only 10 items from the individual domain and 10 items from the team domain were used, with the scores ranging from 6 to 30. The reliability of the scale (Cronbach’s α) was 0.82–0.90 at the time of development and ICC was 0.90 in this study.

#### Clinical performance

Clinical performance was evaluated by developing a scale based on the neonatal resuscitation skills in the order of performance. The scale comprises 22 items, of which 3 were related to the preparation for simulation; 5 to initial intervention; 4 to maintenance of positive airway pressure; 2 to performance of mask, reposition, suction, open airway, pressure, and advanced airway (MRSOPA) drills; 3 to endotracheal intubation; 4 to chest compression; and 1 to wrap-up. The items were scored as follows: 2 for good performance, 1 for moderate performance, and 0 for bad performance. The developed scale was validated by two neonatology professors, one paediatric nursing professor, and two nurses with a master’s degree or higher and at least 5 years of experience working in a NICU. The overall validity was 0.99. As the score distribution differed depending on the process of resuscitation, the average score was used in this scale, and a higher score indicated better clinical performance.

#### Clinical judgement

Clinical judgement was assessed using the Lasater Clinical Judgement Rubric (LCJR) developed by Lasater [19] and translated into Korean by Shin et al. [20]. The LCJR comprises 11 items divided into 4 domains: noticing, interpretation, responding, and reflecting. Each item was rated on a scale from 1 (very poor) to 4 (very good), with a higher score indicating better clinical judgement. The reliability (Cronbach’s α) rates of the tool were 0.88 at the time of development and 0.92 in this study.

#### Interprofessional attitude

Interprofessional attitude was assessed using the Interprofessional Attitudes Scale (IPAS) developed by Norris et al. [21] and translated and modified into Korean by Park et al. [22]. The Korean version of the IPAS comprises 20 items divided into 4 domains: teamwork, patient-centred attitude, respect for cultural diversity, and community-centred attitude. Each item was rated from 1 (strongly disagree) to 5 (strongly agree), with a higher score indicating a better interprofessional attitude. The reliability (Cronbach’s α) rates of the tool for each domain ranged from 0.62 to 0.92 at the time of development, from 0.80 to 0.89 in the study conducted by Park et al. [22], and 0.92 in this study.

#### Education satisfaction

Satisfaction with the simulation-based IPE programme was assessed using 11 items, which were divided into the following domains: education programme, instructor, participant attitude, education duration, and site of education. Each item was rated from 1 (very unsatisfied) to 5 (very satisfied), with a higher score indicating higher satisfaction.

### Ethical considerations

This study was approved by the K University International Review Board (No. 40525-202106-HR-029-04). Written informed consent was obtained from the medical staff for participation in the study and for the results to be published. Participants were informed that they could opt-out at any time during the study and that the refusal to participate would not have any disadvantages.

### Data collection procedures

Data were collected from 1 December, 2021, to 10 March, 2022. The nature, aim, and process of the study were explained to the healthcare professionals working in the newborn nursery or NICU before the recruitment, and 27 participants who signed a written informed consent were enrolled. The study schedule was established based on the physicians’ and nurses’ work schedules after obtaining their consent, and simulation training was performed accordingly. Two rounds of simulation training were provided, with a 2-week interval between trainings. The simulation was performed at the simulation centre of the College of Nursing at K University, and video recordings were made during training for evaluation. Theoretical education was provided online, and the participants were asked to complete the EDpuzzle quiz (EDpuzzle INC, Barcelona, Spain) anonymously. The online education was divided into content related to neonatal resuscitation for the enhancement of interprofessional teamwork, communication skills, and clinical judgement. After completing all lectures, participants were required to take nine quizzes on the lecture content through EDpuzzle.

### Data analysis

The collected data were analysed using SPSS 27.0 (IBM, Armonk, New York, USA), with the following statistical techniques. The general characteristics of participants were analysed using percentage, mean, and standard deviation. Changes in teamwork, communication skills, clinical performance, clinical judgement, and interprofessional attitude after simulation-based IPE programme were analysed using mean and standard deviation and the Wilcoxon signed-ranked test. Finally, satisfaction with the simulation-based IPE programme was analysed with minimum and maximum or mean and standard deviation.

## Results

### Participants’ general characteristics

The participants’ general characteristics are shown in Table 1. The mean age of the participants was 29.26 (± 4.75) years, and the proportion of women (*n* = 23, 85.2%) was higher than that of men (*n* = 4, 14.8%). Moreover, 59.3% of the participants completed a bachelor’s degree (*n* = 16), 33.3% were pursuing a master’s degree or higher (*n* = 9), and 7.4% completed an associate degree (*n* = 2). Overall, the longest work experience was ≤ 2 years (*n* = 9, 33.3%) or 3–4 years (*n* = 9, 33.3%), with a mean length of 4.84 (± 3.6) years. For those working in the NICU or newborn nursery, the longest work experience was ≤ 2 years (*n* = 14, 51.9%), with a mean length of 3.39 (± 2.23) years. A total of 13 participants had prior IPE (48.1%), while 14 did not receive prior IPE (51.9%).

**Table 1:** General characteristics of the participants

(*N* = 27)				
Characteristics	Categories	*n*	%	M ± SD
Age (years)	≤ 25	8	29.6	29.26(± 4.75)
	26–30	11	40.8	
	31–35	4	14.8	
	≥ 36	4	14.8	
Sex	Male	4	14.8	
	Female	23	85.2	
Education	College	2	7.4	
	University	16	59.3	
	Graduate School	9	33.3	
Job	Doctor	9	33.3	
	Nurse	18	66.7	
Total work experience as a nurse or a doctor	≤ 2	9	33.3	4.84(± 3.6)
	3–4	9	33.3	
	5–6	4	14.8	
	≥ 7	5	18.6	
Work experience in NICU^*^ or newborn nursery	≤ 2	14	51.9	3.39(± 2.23)
	3–4	6	22.2	
	5–6	4	14.8	
	≥ 7	3	11.1	
Experience of Interprofessional Education	Yes	13	48.1	
	No	14	51.9	

^*^NICU: Neonatal Intensive Care Unit

SD: standard deviation

### Teamwork, communication skills, and clinical performance

Teamwork, communication skills, and clinical performance were assessed per team (nine teams), while clinical judgement, interprofessional attitude, and education satisfaction were assessed per individual participant (27 individuals). Changes in teamwork, interprofessional communication skills, and clinical performances are shown in Table 2. As the number of participants in the study was not normally distributed, the Wilcoxon signed-rank test was conducted, resulting in a statistical value of *Z*.

**Table 2:** Comparison of teamwork, communication skills, and clinical performance

(*N* = 9)					
Characteristics	Categories	1st simulation (*n* = 9)	2nd simulation (*n* = 9)	*Z*	*p*
		M ± SD	M ± SD		
Teamwork	Total	27.33 ± 3.32	39.11 ± 2.62	−2.67	0.008
	Team	1.11 ± 0.33	1.89 ± 0.33	−2.65	0.008
	Leadership (team leader)	18.44 ± 2.24	25.11 ± 2.20	−2.68	0.007
	Management (team members)	7.78 ± 2.28	12.11 ± 1.62	−2.69	0.007
Communication skills	Total	19.89 ± 3.22	41.22 ± 5.01	−2.68	0.007
	Individual	10.00 ± 2.06	20.89 ± 2.57	−2.67	0.008
	Team	9.89 ± 1.36	20.89 ± 2.57	−2.68	0.007
Clinical performance	Total	0.69 ± 0.09	0.86 ± 0.11	−2.52	0.012
	Initial care	1.49 ± 0.23	1.67 ± 0.23	−2.27	0.023
	PPV	1.47 ± 2.91	1.69 ± 1.67	−1.71	0.086
	MRSOPA	0.50 ± 0.66	1.78 ± 0.36	−2.54	0.011
	Intubation^*^	1.48 ± 0.34	1.56 ± 0.19	−0.58	0.564
	Chest compression				
	Finish care	1.67 ± 0.50	2.00 ± 0.00	−1.73	0.083

^*^The *n* values for endotracheal intubation were 9 at the first simulation and 3 at the second simulation.

*Z* value resulting from the Wilcoxon signed-rank test.

PPV: positive pressure ventilation

MRSOPA: mask, reposition, suction, open airway, pressure, advanced airway

SD: standard deviation

The teamwork score significantly increased from 27.33 in the first simulation to 39.11 in the second simulation (*Z* = − 2.67, *p* = .008). The communication skills score significantly increased from 19.89 in the first simulation to 41.22 in the second simulation (*Z* = − 2.68, *p* = .007).

The average clinical performance score significantly increased from 0.69 in the first simulation to 0.86 in the second simulation (*Z* = − 2.52, *p* = .012). By domain, the score for the initial intervention increased from 1.49 in the first simulation to 1.67 in the second simulation (*Z* = − 2.27, *p* = .023). The score for PPV increased from 1.47 in the first simulation to 1.67 in the second simulation, but the change was not significant (*Z* = − 1.71, *p* = .086). The score for MRSOPA increased from 0.50 in the first simulation to 1.78 in the second simulation (*Z* = − 2.54, *p* = .011). Nine teams successfully performed endotracheal intubation in the first simulation, but only three teams successfully performed this procedure in the second simulation; although the score increased from 1.48 in the first simulation to 1.56, the change was not significant (*Z* = − 0.58, *p* = .564). None of the teams performed chest compression. The score for wrap-up increased from 1.67 in the first simulation to 2.00 in the second simulation, but the change was not significant (*Z* = − 1.73, *p* = .083).

### Clinical judgement

The clinical judgement score and change in the interprofessional attitude are shown in Table 3. To observe changes in clinical judgement after online education, the effectiveness of the programme was assessed based on the participants’ self-reported and instructor-evaluated scores, with separate evaluations conducted to compare each score. Significant improvements were observed in both self-reported and instructor-evaluated scores after the programme. The total clinical judgement scores self-assessed by participant’ significantly increased from 22.74 at the baseline to 36.70 after the programme (*Z* = − 4.52, *p* < .001). The total clinical judgement scores assessed by instructors significantly increased from 30.70 at the baseline to 39.52 after the programme (*Z* = − 4.52, *p* < .001).

### Interprofessional attitude

The total interprofessional attitude score significantly increased from 83.00 at the baseline to 92.04 after the programme (*Z* = − 3.64, *p* < .001).

### Educational satisfaction

Satisfaction with simulation-based IPE programmes was assessed. The total satisfaction score was 52 (± 3.52), with an average of 4.73 (± 0.32).

**Table 3:** Comparison of clinical judgement and interprofessional attitude

(*N* = 27)						
Characteristics	Categories		Pre-training (*n* = 27)	Post-training (*n* = 27)	*Z*	*p*
			M ± SD	M ± SD		
Clinical Judgement	Total	Self-reported	22.74 ± 5.16	36.70 ± 5.13	−4.52	< 0.001
		Instructor-evaluated	30.70 ± 4.61	39.52 ± 3.27	−4.52	< 0.001
	Noticing	Self-reported	6.11 ± 1.76	10.22 ± 1.48	−4.51	< 0.001
		Instructor-evaluated	8.19 ± 1.52	10.48 ± 1.09	−4.05	< 0.001
	Interpretation	Self-reported	4.07 ± 1.27	6.70 ± 1.14	−4.26	< 0.001
		Instructor-evaluated	5.56 ± 0.97	7.03 ± 0.81	−4.09	< 0.001
	Responding	Self-reported	8.26 ± 1.95	13.03 ± 1.87	−4.48	< 0.001
		Instructor-evaluated	10.85 ± 2.20	14.07 ± 1.80	−4.03	< 0.001
	Reflecting	Self-reported	4.30 ± 0.91	6.74 ± 1.02	−4.42	< 0.001
		Instructor-evaluated	6.11 ± 0.64	7.93 ± 0.27	−4.78	< 0.001
Interprofessional attitude	Total		83.00 ± 7.61	92.04 ± 7.40	−3.64	< 0.001
	Teamwork		33.22 ± 2.93	36.85 ± 3.07	−3.22	0.001
	Patient-centredness		17.48 ± 1.70	18.51 ± 1.72	−2.56	0.010
	Diversity and ethics		8.33 ± 1.20	9.11 ± 0.74	−2.37	0.018
	Community-centredness		23.96 ± 3.83	27.56 ± 2.40	−3.81	< 0.001

*Z* value resulting from the Wilcoxon signed-rank test.

## Discussion

In this study, simulation scenarios varied from simple to complex, tailored to the content and objectives of practical training. The team’s doctor demonstrated leadership by understanding situations and executing comprehensive treatments, aiming to enhance the team’s ability to deliver integrated care. Team members were encouraged to perform procedures based on communication with other healthcare professionals, rather than simply executing techniques. To foster teamwork and communication skills, situations were created where all team members had to make decisions together. In addition to essential items for the scenarios, various airway devices, laryngoscope blades, ambu bags, and masks of different sizes were prepared. Placing unnecessary items alongside those required prompted participants to select the appropriate ones, thus encouraging critical thinking. This aligns with prior literature suggesting that critical thinking improves clinical decision-making abilities [23].

The programme comprised simulation and theoretical learning, with online lectures provided. While lectures efficiently deliver knowledge, they may not foster critical thinking, problem-solving, and creativity. To address this, EDpuzzle, an interactive online content platform, was utilised alongside traditional lectures. EDpuzzle engages students by incorporating video-watching and quizzes, enhancing concentration, confidence, and self-directed learning skills [24]. Positive feedback was received regarding the use of EDpuzzle, which compensated for the individual online learning necessitated by COVID-19 and facilitated the cultivation of critical thinking.

Effective teamwork and leadership are crucial for neonatal resuscitation. Theoretical learning included content on teamwork and communication skills, while simulation included prebriefing and debriefing. During debriefing, a reflection process on procedures was added, along with practicing and recording the SBAR communication method learned in theoretical learning. This provided an important opportunity to practice communication skills necessary in clinical settings and compare communication styles among those who experienced the same simulation.

In this simulation-based IPE programme, theoretical learning was conducted through online education without constraints of time and space, utilising EDpuzzle to enhance interaction between instructors and learners. Furthermore, the programme targeted physicians and nurses in the medical field, differentiating itself from most neonatal resuscitation education in Korea, which primarily targets nurses or nursing students.

This study found that after completing the simulation-based interprofessional programme, teamwork significantly improved among healthcare personnel working in a NICU and Nursery unit. This finding is consistent with those of previous studies reporting improved teamwork after IPE [4, 25]. The simulation was conducted in two rounds using the same scenario and teams, and repetitive learning was applied. Exposure to the simulation served as an opportunity to practice teamwork, which may have led to the improvement in teamwork. The scenario was structured to provide neonatal resuscitation for an extremely preterm, 25-week-old, very low birth weight infant. In the second round of simulation, the participants were at ease, and a more relaxed environment was created, thus contributing to improved teamwork; a relaxed learning environment increases the learners’ participation and motivation [26]. In addition, teaching the importance of teamwork and methods to improve teamwork during theoretical education between simulation sessions possibly contributed to the improvement in teamwork.

After the simulation-based interprofessional programme, communication skills were significantly improved; this finding was similar to that of previous studies, which reported improved team communication after IPE [27]. In the second simulation, more participants used closed-loop communication, which may be the result of learning closed-loop communication and SBAR communication during theory education. Moreover, our results are in line with those of previous studies, which indicated that closed-loop communication is helpful during emergency situations or resuscitations [3, 28]. During the first simulation, most of the physicians provided only orders, while the team members were not involved in the decision-making process. In the second simulation, the physicians encouraged the team members to participate in the decision-making process, and they discussed the status of the infant and determined the direction of treatment as a team. Similarly, enhancing the effectiveness of learning through repetitive learning and exposure and fostering a relaxed environment was helpful.

After the simulation-based interprofessional programme, there was a significant improvement in the clinical performance of the learners, consistent with findings of previous studies [29, 30]. In the first simulation, all nine teams improperly performed MRSOPA and thus had to proceed to endotracheal intubation. In the second simulation, six out of nine teams managed to resuscitate the neonate before endotracheal intubation. Consequently, the MRSOPA score markedly improved from 0.50 in the first simulation to 1.78 in the second simulation. Furthermore, the number of teams that performed endotracheal intubation decreased from nine to three teams; as the non-performance of endotracheal intubation indicated that resuscitation was quickly achieved with only positive airway pressure and MRSOPA, this finding was interpreted as improvement in clinical performance. As endotracheal intubation—an invasive respiratory support—increases the risks for neonatal bronchopulmonary dysplasia and ventilator-related pneumonia and mortality compared with noninvasive respiratory support [31, 32], improving the learners’ clinical performance in the MRSOPA process was a strength of this programme.

Safe and quality treatment and care can be provided with effective teamwork; even skilled and competent healthcare professionals cannot achieve good outcomes when communication is hindered [33]. This study showed that enhanced communication leads to improved teamwork and ultimately to improved clinical performance, an index of team performance. Thus, as a benefit of IPE [13], this simulation-based IPE programme contributed to the improvement of teamwork, communication skills, and clinical performance of the resuscitation team.

After the simulation-based IPE programme, clinical judgement significantly improved. This finding is similar to that of previous studies which reported that simulation education improved the learners’ clinical judgement [34, 35]. Among the clinical judgement domains, the responding domain markedly improved; this domain consists of a confident attitude, clear communication, well-planned interventions, and proficiency of skills. Understanding the scenario, re-assessing the process of solving the patient’s problem, and reflecting based on the feedback provided during the debriefing process contributed to the improvement in clinical judgement. This finding is in line with those of previous studies, which reported that briefing and debriefing can enhance the knowledge and skills of the healthcare staff [36]. Clinical judgement was assessed based on the participants’ self-reporting; we further assessed the participants to obtain more accurate study data. The responding domain obtained the highest score, followed by the noticing, reflecting, and interpretation of both self-report and author-evaluated scores domain; this is consistent with the results of previous studies [37, 38]. Participants were encouraged to assess their own strengths and weaknesses through self-evaluation, and instructors provided feedback based on their evaluations during debriefing sessions. This enabled participants to recognise their abilities relevant to real patient situations and gain a better understanding of their abilities through external evaluation, allowing them to focus on improvement [39].

After the simulation-based IPE programme, interprofessional attitude was significantly improved. Learning about the benefits of IPE and experiencing collaboration through two rounds of simulation possibly contributed to this result. This finding is in line with those of previous studies, which reported that interprofessional collaboration and education can improve the attitude and perception of the healthcare staff [1].

The mean education satisfaction score In this study was 4.73 (± 0.32) out of 5. Using a high-fidelity simulator to achieve more realistic training and creating an environment that closely resembles that of the hospital increased participants’ satisfaction levels. Furthermore, our education was provided in three sessions, with each session lasting 40–50 min; the short duration of each session probably reduced the participants’ burden. Moreover, utilising a new learning instrument called EDpuzzle increased the participants’ engagement in the learning process, thereby increasing their satisfaction.

The objectives of online education were set according to Bloom’s taxonomy. Our education programme improved the participants’ teamwork, communication skills, clinical performance, and clinical judgement, which satisfied the objectives of the psychomotor domain. Improvement in the interprofessional attitude satisfied the objectives of the affective domain. However, we did not assess the cognitive domain; hence, we could not assess whether the objectives of this domain were satisfied. Therefore, follow-up studies should examine whether simulation-based IPE alters the participants’ knowledge, a parameter used to assess the cognitive domain.

Based on the Kirkpatrick model for training evaluation, we assessed the participants’ reaction, which is the first level, to the training by conducting an education satisfaction survey. The mean education satisfaction score was 4.73 out of 5, showing that the participants were highly satisfied with the overall programme. The second level, learning, was divided into attitude and skills. Interprofessional attitude as well as teamwork, communication skills, clinical performance, and clinical judgement were improved after the programme. As both attitude and skills were improved after the programme, the goals in this level were met. However, levels 3 (behaviour) and 4 (results) were not evaluated. According to a theoretical framework for IPE, health outcomes can be improved only when the concepts learned through IPE can be applied in the workplace. Therefore, additional studies are needed to determine how our simulation-based IPE programme is utilised and its impact in an actual clinical setting, and to longitudinally examine the effects of the programme on patient safety and quality of neonatal care.

This study developed an IPE programme based on the theoretical framework for IPE proposed by the WHO [13] and implemented for physicians and nurses. Our results showed that teamwork, communication skills, clinical performance, clinical judgement, and interprofessional attitude were significantly improved after the programme. The objective of IPE is to ensure that collaborative practice becomes a fundamental component of both the education and professional activities of all healthcare personnel, embedding it within the training and delivery of all relevant health services [13]. Consequently, there is international consensus that health professional students need to engage in IPE to prepare for their practical work [13]. With this study confirming the positive effects of IPE, it is hoped that it will be utilised not only by healthcare professionals but also by healthcare students.

### Limitations

Despite the positive effects of our education programme, this study has a few limitations. First, our study included only physicians and nurses recruited from a university hospital in one region; therefore, the findings have limited generalisability. Second, this study was designed as a one-group, pretest-posttest experimental study; hence, exogenous variables possibly influenced the results of the programme. The results of this study should be interpreted with caution. Finally, we could not confirm whether the objectives in the cognitive domain were satisfied per Bloom’s taxonomy or determine the behaviour and results of the Kirkpatrick model. Hence, additional studies are warranted to address these issues.

## Conclusions

After participating in the simulation-based IPE programme, the resuscitation team’s teamwork, communication skills, and clinical performance significantly improved. The participants’ clinical judgement skills and interprofessional attitude improved, and they showed high education satisfaction. Based on these findings, a simulation-based IPE programme is an effective educational method to improve teamwork, communication skills, clinical performance, clinical judgement, and interprofessional attitude. Neonatal resuscitation is the initial step to the healthy survival of neonates; hence, healthcare professionals must enhance the competency of the resuscitation teams so that they can efficiently and effectively perform the necessary skills. Modifying and adapting this programme for repetitive training could help ensure more accurate neonatal resuscitations, ultimately contribute to the reduction of neonatal mortality rate from asphyxia, and enhance the quality of neonatal care.

## Data Availability

The datasets used and analysed during the current study are available from the corresponding author on reasonable request.

## Abbreviations

GAS: Gather-Analysis-Summarise
IPAS: Interprofessional Attitudes Scale
IPE: interprofessional education
IUSIR: Indiana University Simulation Integration Rubric
LCJR: Lasater Clinical Judgement Rubric
MRSOPA: mask, reposition, suction, open airway, pressure, and advanced airway
NICU: Neonatal Intensive Care Units
PPV: Positive Pressure Ventilation
SBAR: Situation-background-assessment-recommendation
STAT: Simulation Team Assessment Tool
WHO: World Health Organization
